# The American Horse

**Published:** 1880-07

**Authors:** George B. Loring

**Affiliations:** Member of Congress from Massachusetts; Salem, Mass.


### THE ARCHIVES
OF
### COMPARATIVE MEDICINE AND SURGERY.

VOL. I. JULY, 1880. No. 3.

### Original Articles, Selections, and Translations.

### ART. I.—THE AMERICAN HORSE.

ADDRESS DELIVERED IN CONNECTION WITH THE COMMENCEMENT EXERCISES OF THE COLUMBIA VETERINARY COLLEGE, AT CHICKERING HALL, NEW YORK, MARCH 26TH, 1880.

BY GEORGE B. LORING, M.D.,
MEMBER OF CONGRESS FROM MASSACHUSETTS.

GENTLEMEN—The modern attempt to elevate veterinary science above the mere empiricism which characterized it universally before the beginning of the present century, and which characterizes it too extensively now, is entitled to profound gratitude and liberal encouragement, on the score of both economy and humanity. Dedicated as this college is to the development and care and preservation of all that portion of the animal kingdom which man has subdued and devoted to his prosperity and comfort, and which constitutes more than $1,500,000,000 of his property in this country alone, it deserves the support of all who are engaged in the practical affairs of life; and the sympathy and encouragement of all who would ameliorate the animal suffering which man forces his dumb allies to share with him in the warfare of life. As I consider what it may do to benefit both man and animal, and the difficult task it has to perform in interpreting voiceless complaints; in finding the seat of pain where no intelligence points the way; in groping for symptoms and diseases amidst the darkness of uninspired animal life; in arresting destroying disease where no enlightened observation points out the destroyer—I am tempted to deal with the relations which exist between man and animals, and to explore that mysterious problem which intelligent thinkers propound for that part of creation whose thoughts and reason are hidden in that solemn and silent realm occupied by instinct alone. But my inclination, in view of the practical object of this college, leads me to the companionship, rather than to the abstract, contemplation of the animal kingdom; and to an intimate association, for an hour at least, with that faithful and fascinating servant, without whom many of our active industries would cease, and our keenest pleasure would be destroyed. While I recognize the value and importance of all other domestic animals, and remember that the value of the cattle of the United States is estimated at nearly a thousand millions; that their annual product in meat is $398,956,000, and in the dairy $187,000,000—$9,000,000 more than our product of cotton goods, and $26,-000,000 more than our woolen goods—I cannot forget the value of the horses of our country, or close my eyes to the fact that the relations which exist between man and the horse are of such an intimate and significant character that they cannot be destroyed or violated without producing an effect deeper than that produced by the simple loss of property. Somehow, the horse has managed to connect himself with so much that is interesting and valuable in life that we cannot abuse or insult him without wounding our self-respect; we cannot destroy him without serious loss. He occupies a strange and important place in our history. In great military expeditions, he has always performed an important part. Old warriors used him. Old scholars wrote about him. Jacob commenced early trading corn for horses with the Egyptians, and a long array of chariots and horses followed this patriarch in funeral procession. He was an Egyptian animal at a time when Egyptian civilization outshone all others; and I am of opinion that he has found his most congenial companions where cultivation and refinement have prevailed. From the days of Pharoah until now, as the arts of life advance, how he goes with them! I find him in Arabia the ally and protector and companion of man—his best possession there. I find him immortalized in the finest marbles of Greece and Rome. I find pages in history dedicated to the record of his wonderful deeds on the turf, on the road, at labor, in the chase, and on the field of battle. Kings have devoted the royal treasury to his increase, improvement, and comfort; and ambitious and enthusiastic agriculturists have applied themselves unsparingly to his introduction into the best regions and systems of farming. Why, what a flood of charming associations and memories rushes around us, as we recall the position which the horse has held for almost all time! William the Conqueror and his Roman horses; King John and his Flemish horses; the admiring crowds that gathered around the Darley and the Godolphin Arabian; the enthusiastic admirers of Sir Auley and Sir Charles; of Lexington and Boston; of old Eclipse; the stables of Washington and the thorough-breds of Jefferson. It is not worth while to tell me that there is nothing more in all this than the simple ownership of so many mentionable animals, to be valued by weight in the market. In great events of joy and sorrow, in crises and revolutions, the horse finds his place, standing next to man, the partner of his fortunes and his fate, and performing an important part in all the drama. I have been so struck with the place assigned the horse, in all the stirring incidents of chivalrous personal history, that I remember always the touching lines, which is the introduction to "The Betrothed," tell the vision which descended on the "Noble Maringer":—

> "They tower and their banner knew thy steed and thy rein,
> And stoop them to another's will thy gallant vassal train;
> And she, the lady of thy love, so faithful once and fair,
> This night, without thy father's hall, she weds Marstettin's heir"

Towers, horse, vassals, and lady-love—all join to make the significant picture. But not in deeds of war and chivalry alone has the horse endeared himself to man. I have said he seems to belong, by right, to the highest civilization, and to find there his most favoring and congenial home. Not, however, to this sphere alone is his genius confined. Obedient to surrounding circumstances as no other animal seems capable of being, his frame and temperament alike conform to the necessities of which he meets. The pride of the race-course, to which he is often led when he is but two years old, prematurely developed by protection and care into all the nerve and vigor of mature life, restless, impatient, and beautiful, he finds an elephantine, stolid, patient brother leaving the pastures of Holland and the Clyde for the weary toil of the brewery and the coal-yard; he finds a hardy, diminutive, busy, cool, and sagacious member of his family browsing on the ferns and moss of the Orkneys; he hails from the desert the lithe and sinewy form of a more immediate relative; he looks quietly on as his self-poised American cousin whirls along the road with that tremendous stride which has been developed by the wants of a free and driving people, each one of whom is bound to reach his destination first; and he finds a rough and wiry specimen of his race scouring his plains in all the vigor of savage life, proving his characteristics under all circumstances, and in whatever form he may appear. He gradually adapts himself to soil and climate and circumstance with a readiness unknown to any other animal but man. In the battle-field, he is a war-horse; on the race-course, he is a deer; on the farm, he is a drudge; on the road, he is a locomotive; at the civic procession, he is as airy as his rider; as a hack, he is sagacious in the use of his forces; at the stage-coach, he is "flying all abroad;" at the private carriage, he is as proud and disdainful as the petted beauty who sits behind him. It is in this cosmopolitan animal family that the American horse takes a high place. Not in any sense a thorough-bred trotter, as he is sometimes called; for that name belongs to a breed of horses as distinct under this name as the Arab or the Barb is under his name. No Englishman speaks of a thorough-bred as a thoroughbred Orloff, or a thorough-bred Canadian, or a thorough-bred Persian. The thorough-bred is, in his mind, a distinct breed of horses, and the term belongs to no other. The name does not belong to cattle, for we have no such breed of cattle. We have Shorthorns and Ayrshires and Devons and Herefords, but no thorough-breds as a breed. It is as manifestly improper to apply the name thorough-bred to a known and named breed of cattle as it is to another and varied breed of horses. You cannot speak of thorough-bred Shorthorns more than you can speak of thorough-bred Arabians. The names of Shorthorns, Ayreshires, Devons, Herefords, among cattle, are sufficient, without any prefix. But if you desire especially to indicate the purity of your cattle with marked distinctness, you can call them pure-bred Shorthorns, etc., but not "thorough-breds." Not in any sense is the American horse a thorough-bred; but he is an animal after his kind, and unequalled by any other horse on the face of the earth in all that makes such an animal truly valuable in every kind of service. It takes true equine genius to make what is known as the American horse—that animal which, when he has reached the height of his faculties, is known as the American trotting horse. His mechanism must be as well-balanced and symmetrical as a locomotive. Propelled as he is by one quarter at a time, his progress is the result of nerve and strength and decision unknown, and utterlly ignored, in that leaping, bounding motion of the running horse of the English turf. The American horse must be solid on the foot, strong in the limb, firm in the back, free and easy in his stride, and, above all things, calm and collected amidst all trials of the track and the road, which tend to throw him off his balance, and reduce him to the level of the hare and the fox and greyhound—running helter-skelter, without the exercise of any faculties except those with which nature endows him who flees from danger or conflict. The American trotter requires bones and muscles and brains, and when he stands high on the list, he has them all. For compactness of form and ease of motion; for strength and endurance and sagacity, he is unequalled. Now, this animal we have almost as the natural product of our farms. Descended from the thorough-breds of generations long since gone, he is undoubtedly, as he *now* appears, the result of that social and civil equality which, in our country, makes one man's time as valuable as anothers, and which authorizes the farmer's boy to take the road from the parson or the doctor whenever he can get it.

Every man in this country who can keep a horse wants a good one; and, when he has got him, he wants to avail himself of his horse's powers to make the distance between one place and another as short as possible. We all drive on the road; and this continued, with certain fortunate aptitudes of soil and climate, has given us all the peculiar merits of the American horse, heir as he is to strong physical power and tractable domestic faculties. Why, then, should we go abroad with the expectation of improving what we now have? While we have the many valuable families well known to us, so divine in size and shape, so well-fitted by form and temper to every labor, and yet possessing a kind of prevailing uniformity expressed by the term "a horse of all work"—can we hope to derive much benefit from a resort to those specific breed of horses which, in England, are devoted each to its own specialty? There is no necessity, for instance, for importing a Suffolk branch; for half a day's search would undoubtedly provide you with just such an animal, raised on your own soil, which, in all its varieties, develops almost every style and shape and quality of horse known on earth. We need not import hunters, for we have no need of such a horse among us. The Cleveland bay, valuable as a carriage horse, could hardly expect to improve the stylish breeds of the South and West.

The adventures of the thorough-bred of America on the English turf shows our capacity for producing that class of animals; and when we consider that it is only after we have reached many removes from the thorough-bred that we have arrived at good trotters; when we remember that neither in shoulder, nor leg, nor quarter, nor general mechanism, is there any analogy between the thoroughbred as raised in England, and the trotter as raised in our own country—we may well ask ourselves what advantage is to be derived from the introduction of such animals among us? In saying this, I do not fail to recognize the value of those old progenitors who brought into our country, many years ago, the bone and muscle and nerve and wind and capacity of the English thorough-bred of that day. I am mindful of old Messenger, and of what he and his sons have done; and I cannot forget that his fame, as the ancestor of trotters, was established not in Bucks County, Pa., but on Long Island, and various other points in the State of New York, whence his stock was distributed throughout all the fair, hilly, breezy, brainy, horse-growing sections of New England. As the sire of Miller's Daniel, and of Sir Harry (thorough-breds), he won a fair reputation; but it was as the sire of Mambrino, whose dam and grand-dam were of unknown blood, and not allied to the thorough-bred, that he won his distinction as the ancestor of some of the most remarkable trotters known on earth; and how, as generations went on, and that unknown blood worked indeed the spread of his family increase. From Mambrino sprung Abdallah, dam Amazonia, and Mambrino Paymaster, with unknown dams and great accomplishments.

From Abdallah, with his unknown grandmother, we have two or three generations removed, each with its *unknown* dam, Rysdyk's Hambletonian, with his famous sons, Dexter, George Wilkes, and Mountain Boy. From Mambrino Paymaster, with his unknown dam, we have Mambrino Chief; we have Lady Thorne and Mambrino Pilot and Mambrino Patchen and Erricsson and Ashland, in whose pedigree will be found as many unknown dams as there are sires and grandsires. And, as I trace the blood of old Messenger into Maine and Vermont, where all the dams were *unknown*, what a tribe of our earliest and best trotters rises before my vision! Ripton, the gallant "white-legged pony," the favorite of Hiram Woodruff, the resolute and triumphant, revelling Dutchman, as a three-miler, and defeating Lady Suffolk—an Eastern horse of undoubted Messenger and Morgan blood; and Daniel D. Tompkins, a wonderful little horse; and Gen. Taylor, a very fast trotter and sticker; and Independence, the delight of my boyhood; and Fanny Pallen, Green Mountain, Maid and Gray Vermont; and Ethan Allen—the best balanced horse ever seen on the American track; the easiest-gaited horse, from the Walkormund, ever bred, and the most striking illustration of the enervating influence of high feed and rapid work in early life ever known in horse annals. These horses, far removed from the original thorough-bred, and fortunate in the strain of blood which they do possess, springing from families in which the admixture of various races is undoubtedly to be found, members of a list honorable and illustrious, commencing with Topgallant and Whalebone and Dutchman and Confidence and Washington and Rattler and Lady Suffolk, with their *unknown* strains, and ending in our day with Flora Temple and Goldsmith Maid and Dexter and American Girl and Lucy and Bonner's Pocahontas (the queen of mares), with their great records, and their absolute defiance of time and space. These horses, I say, illustrate what I mean by the power of the American trotter, which is to be obtained by removal, step by step, from the form and gait of the thorough-bred. Hence, then, our American horse. A keen, sharp driver among our sweet northern hilly pastures, cold winter and crystal springs; a heavy draft-horse on the more luxurious grazing in the milder climate of the Middle States; a Clydesdale here and a thorough-bred there, with all the diversity of nature which marks the great territory of the United States, which includes so many climates and varieties of soil, born to every variety of toil and to every variety of influence.

And so we have the American horse all along the northern line, from Eastport to Detroit, or still further West—a fortunate combination of various blood, invigorated by the sharp air of our Northern hills, refreshed by our cold Northern streams, formed into hard bone and vigorous muscle, and capable of implanting his sturdy forms among the heavier bones and softer muscles of more luxurious valleys, milder skies, and warmer springs.

That he gets somewhat of his power from his native soil and climate there can be no doubt. But how has he converted that stilted gait of the thorough-bred into the swinging stride and powerful knee-action of the trotter? What has changed the narrow and confined shoulder of the thorough-bred, with its short humerus attached, and the necessarily advanced position of the fore-leg—so near the point of the shoulder that a line falling thence touches the toe—to loose shoulder-blade and long humerus; long from the elbow to the point of the shoulder, so that a line falling from this point touches the ground far in front of the foot, and to that massive, muscular base which characterizes the trotter?

What has cut down the sharp, thin withers of the thorough-bred, and filled in the space above the top of the shoulder-blades with a mass of strong muscles? What has strengthened the lower jaw, so that horse and rider may be made one through bit and rein? What has dropped the points of the hips below the level of the rump, where they stand usually on the thorough-bred? What has judiciously cooled the ardor and increased the patience and enlarged the sagacity of the thorough-bred? What has encased the untiring channels of true blood in a new frame, of proportions hitherto unknown to them, until they were subjected to the influence of American companions, American wants, and American institutions? Probably no single cause, but many combined. The habit of driving to which I have alluded has done much toward bringing about this result. But this alone is not sufficient, and I am constrained to believe that we owe much of the shape and stride which distinguish our best trotters to a larger or smaller infusion of Canadian blood, derived from the early importations of Norman horses into Canada, which have been improved in size and quality by the soil and climate of their new home. In very many of our good trotters, this is manifest. All the descendants of Henry Clay, whose dam was Surry, a mare of great speed from Canada, have the thick jowl and heavy ear and round muscles and coarse-grained foot of the family from which they sprung.

But I want to say a word about the care and management of this valuable animal.

When a colt is born into a family—especially if his lot is cast in pleasant places, and he has a goodly heritage—the foremost danger is that he will be spoiled in early life. It really seems as if all owners of horses endeavored to ascertain how in the most expeditious manner to ruin them.

The natural tendency of a horse, young or old, is to preserve himself in a sound and healthy condition. The wear and tear of hard work, and the injurious effects of a life of luxury and ease, are about equally destructive to him; and the price he is obliged to pay for his intimacy with man, and the care and attention he receives at his hands, is the loss, in a larger or smaller degree, of the robust health, and the elastic animal spirits, and the abounding and joyous and painless power of motion with which nature endows him. A colt is a happy thing in the beginning—happier than a child. A horse is intended to be a happy thing through life—happier than man. But the folly and misfortune which sadden and weaken the master bear heavily, also, on his dumb and patient servant. The two travel a hard road together, and both are obliged to pay the penalty which should in justice fall upon one.

If this is one of the inevitable consequences of the decree which gives man dominion over the birds of the air and beasts of the field, I suppose man and animal must submit and obey. But it may not be so. If, for the gratification of ambition or pride, or for high service to his race, or for immortal renown, man is willing to subordinate and sacrifice all his physical powers, and is determined that his body shall obey the commands of his imperious spirit, inspired and consumed in the great flame, so must it be; but let him spare his servant who obeys him, his dumb beast who has trusted in him.

It is a good thing to remember that a horse has certain natural faculties, without which he would not be a horse, and which it is important to preserve. Man is so wise as well as tyrannical that he finds it difficult to believe that he is not to remodel and reconstruct everything which is provided for his use and comfort before it is fit for his imperial use and service; and so he meddles with everybody and everything. It is much easier for him to comprehend his own handiwork than the Lord's. His boy, for instance, stands before him a bright, strong, attractive lad, full of capacity and promise; a combination of faculties, good and bad, each striving for supremacy; a fresh and glorious creation from the hand of God, intended to rejoice his father and bless mankind. It is only necessary for that father to know when to encourage him, when to suppress him, and when to let him alone, to distinguish between healthy powers, which a superabundance of youth and strength may sometimes make effusive, and those unhealthy deformities, which, even while quiet and slumbering, are disgusting and discouraging. But this is no easy task. When there should be mutual confidence, a contest begins, and before it ends the boy has lost his self-respect, his love, his confidence in his fellow-men; his virtues are discouraged, his vices rage, or, it may be, that in rooting up the tares the wheat has been pulled up along with them, or his good points may have been distorted into subserviency and inefficiency, while his bad ones may have learned how to play the hypocrite. When, too, there should be a manly and dignified intercourse, there is too often an effeminate and enervating intimacy. The boy may be softened into abject dependence upon those who should inspire his most manly self-reliance. That apron-string business—how many a brave fellow has it sent mewling through life like a milk-sop!

His father has made a good boy of him, but not the boy he was intended to be. The problem has been solved, but not in the right way; and in the trials which follow, he wonders where those qualities are which he felt moving within him in his youth, and the father wonders why he is so little satisfied with the work of his own hands.

No, do not bother or confuse the boys. Do not meddle with them too much. Make them way-wise early. Do not pet them into weakness, or curb them into madness; and when they go forth into life, let them have manliness to meet their fellow-men in a manly way; generosity enough to warm a generous feeling in the breasts of their associates; charity enough to forgive the faults of their fellow-men; and humanity enough to know that it is better and more useful to encourage the virtues than to expose the vices of society, and more honorable to set a good example than to pronounce a good precept.

But to return to the colts. They, like the boys, may be spoiled by too much meddling. They should neither be petted to death, nor conquered and subdued to death. They should be familiarized with the harness when so young that they may imagine the straps a part of themselves. They should never know what it is to be broken. They should find themselves engaged in business they hardly know how; and they should be gradually introduced to their work with an unruffled temper, and an acquiescent but unsubdued spirit. When you actually conquer a horse, you can never tell where the conquest is going to end. When Flora Temple was conquered, and her unruly spirit was broken and not improved, she was worthless. When she was taught to know herself, she was invaluable. Let the lesson begin at two years old, and a few weeks will complete it, without danger from violence, and it will never be forgotten. It need not be renewed until the animal has become mature and strong enough to bear the burdens of life. As a colt may be sprained by over-loading, so he may be ruined by over-feeding. I have known many a colt ruined by excessive feeding the first winter of his life. It is pleasant to see his glossy coat and lively head and mature neck and well-developed form, under a good supply of oats. But all the pleasure will vanish if you look carefully at his limbs, which tremble a little after exercise; and it will still more entirely vanish if you will examine him after his summer's run at grass, and wonder that he looks no better, and has not grown more. Sweet hay and a few oats, with a very little grain at long intervals of time, are sufficient. And if you doubt this, go and ask Ethan Allen and hundreds of his descendants, who went through the enervating process of high feeding when young; go and ask the fat and favorite colts who are passing their hot-bred lives in their good-looking stables, which are multiplying everywhere; go and ask the thousands of English thorough-breds, who are hobbling about, ruined by forced growth, and forced efforts, and hot food, ere their lives had fairly begun, and see what answer you will get. They would all tell you, if they could speak, that the muscle which the horse gets after he is four years old is worth vastly more than what he makes before that time; that all the fat a colt had upon himself before he is four years old is an injury to him; that their life is shortened and their powers are weakened by early feeding and early work; that if you will feed for early maturity, and drive for early speed, you must expect to lose a large part of the ultimate value of your horse. A few years of life, a few seconds of speed. Precocity is a poor thing. That alone endures which ripens slowly. The wisdom of human maturity is the best wisdom, that maturity which comes from the steady and legitimate development of all human powers. That speed and endurance are the greatest which are not called for until the horse is in full possession of his faculties. An American man, dependent on himself for all he is and is to be, fit for all the duties which may devolve upon him, will not grow up in a day.

An American horse of all work, destined to toil like a locomotive, and expected to travel like one, wants time to develop for his tremendous services. Of the management of the horse I have but little more to say, but that little is important to all who are studying veterinary science for practical use. Avoid, then, hot and enervating stables for the young to grow in and the mature to rest in. Never allow a horse to stand on a wooden floor; bricks are better. Many diseases of the feet are brought on by the drying and heating influence of wood. Reform, if possible, the situation of all stables into which horses fresh from the pure air of the country are crowded and stifled and ruined. Remember, it is easier to preserve the health of man and animals than it is to restore it when lost. Beware of too free use of drugs. A horse is very sensitive to the influence of medicinal agents—much more so than a man, and is often longer in overcoming the effects of medicine than the effects of disease. If he becomes unsound, give him instant rest, affection, and immediate treatment, and never encourage yourself with the assurance that the unsoundness is in a comparative safe place, when there is every indication that it is not—that it is in the shoulder, where it seldom is, and not in the foot, where it too often is. Pardon me for this practical advice; but I have paid a large price for my experience, and should be very sorry to have the world lose the benefit of it; and I am sure every scientific investigator into the profession which you are studying here, desires to see his science rest where Lord Bacon placed all true science—*on the solid foundation of common sense*

I have on this occasion discussed in a practical manner the American horse, because I desire not only to appeal for his welfare, but also to secure for him proper consideration as one of the noblest and most useful of our servants. We all appreciate him, I know, but we do not all understand him, and we shall not have reached the highest point of a humane and economical organization until we bestow upon the horse the *same care* and kindness in his hard toil which he shares with his master, as we bestow on him when he performs his part as an aid to our comfort and luxury and pleasure. He has done his duty well thus far, and shall we not do our duty by him. The value of his work cannot be over-estimated. Deduct all that has been achieved, directly or indirectly, by the aid of the horse in the way of conveyance from place to place, for business or recreation, for distant journeyings, before the power of steam was so wonderfully applied to the purposes of locomotion; of the draught and heavy burdens of motive power connected with machinery, of agriculture, and of war in all countries and in all ages; deduct all that has been done, directly or indirectly, in all these respects, by the aid of the horse, and what a stupendous abatement you would make from the sum total of achievement and progress. And now allow me to repeat an interesting and touching account of the relations which existed between one of the most illustrious of our own race and one of the most fortunate of that race for whose cause I am speaking. How well I remember it as it fell, not many years ago, from the eloquent lips of Edward Everett! After urging for the horse persevering kindness, and asking if this would not also be beneficial and honorable among fellow men and fellow Christians, he said: However this may be, if there is any one who doubts that the horse, the animal that most concerns us on this occasion, is susceptible of the kindest feelings of our nature, I think he would be convinced of his error by a most interesting anecdote of Edmund Burke. In the decline of Mr. Burke's life, when he was living in retirement on his farm at Beaconsfield, the rumor went up to London that he had gone mad, and the fact that was stated in support of this rumor was, that he went round his park kissing his horses and cows. A friend, a man of rank and influence, hearing the story, and deeming it of too much importance to be left uncorrected, hastened down to Beaconsfield and sought an interview, with the view of ascertaining the truth of the rumor: He entered into conversation with him. Mr. Burke read to him some chapters from his "Letters on a Regicidc Peace." His friend immediately saw that though his earthly tenement was verging back to its native dust, the lamp of reason and genius shone with undiminished lustre within. He was accordingly more than satisfied as to the object of his coming down, and in a private interview with Mrs. Burke told her what he had come for, and received from her this pathetic explanation: Mr. Burke's only child, a beloved son, had not long before died, leaving behind him a favorite old horse, the companion of his excursions of business and pleasure, when both were young and vigorous. The favorite animal was turned out by Mr. Burke, the father, into the park, with directions to all his servants that he should in every respect be treated as a privileged favorite. Mr. Burke himself, of course, in his morning walks, would often stop to caress the favorite animal. On one occasion, as he was taking his morning walk through the park, he perceived the poor old animal at a distance, and noticed in turn that he was recognized by him. The horse drew nearer and nearer to Mr. Burke, stopped, eyed him with a most pleading look of recognition, which said as plainly as words could have said, "I have lost him, too," and then the poor dumb beast deliberately laid his head on Mr. Burke's bosom.

Struck by the singularity of the occurrence, more by the recollection of his son, whom he had never ceased to mourn with a grief that could not be comforted; overwhelmed by the tenderness of the animal, expressed in the mute eloquence of holy nature's universal language, the illustrious statesman for a moment lost his self-possession, and, clasping his arms around the neck of his son's favorite animal, lifted up that voice which had filled the arches of Westminster Hall with the noblest strains that ever echoed within them, and wept aloud.

This was seen and heard by the passers-by, and the enemies of Burke, unappeased by his advancing years, by his failing health, by his domestic sorrow, made it the ground of a charge of insanity. "Burke has gone mad." But, so help me heaven, if I were called upon to designate the event or period in Burke's life that would best sustain a charge of insanity, it would not be where, in the gush of the holiest and purest feeling that ever stirred the human heart, he wept aloud on the neck of his dead son's favorite horse; but it would rather be at the meridian of his fame, where the orb of his imperial genius rode highest in the heavens, amidst the scoffs of cringing courtiers, and the sneers of trading patriots, he abused his glorious powers to the scramblings and squabblings of the day, and,

> "Born for the universe, narrowed his mind,
> And to party gave up what was meant for mankind."

And now, gentlemen, you will allow me to discuss, in conclusion, this institution, which is dedicated to the welfare of that animal kingdom which man has subdued for his own comfort and convenience, and perhaps for his very existence.

As one of the pioneers in veterinary education in this country it is entitled to all the encouragement which can be bestowed upon it by all who believe in scientific investigation, into the laws of animal life for the preservation of health and the removing of disease.

To the improvement and economical protection of nearly 40,000,000 of dumb animals, man's servants and allies in the work of life, representing property to the amount of $1,000,000,000 at least, this college is dedicated, and it seeks to apply to the animal kingdom all the laws of therapeutics and hygiene which are applied to its owner and master. It would be impossible to estimate the amount of suffering caused in this country by mal-practice on sick and diseased brutes, whose pangs are often increased rather than relieved by harsh remedies and their harsher administration.

No dumb animal has yet told the long tale of agony growing out of the rude surgery which has been applied to the race of his generation.

The loss of property arising from bad management, exposure to poisonous gases, supply of bad food, insufficient care, brutal abuse, wasting our pastures and stables, can hardly be calculated.

Upon an institution designed to inform the humane as well as the thrifty owners of horses and cattle how they may remedy these evils, I look with especial favor.

The application of scientific skill to the preservation of soundness and health, and the curing of disease among the vast number of valuable working and driving horses in this city alone—can you over-estimate its importance?

The state and national councils of this country are engaged at this very hour in devising means by which the destructive epidemics which destroy our herds can be stayed, and finally removed, and the great want, as I well know, is a corps of well-educated practical veterinary surgeons, skilled in the treatment of disease and the laws of health, capable of defining how much is disease and how much a popular panic; wise to advise the most economical methods of curing or of isolation and extirpation, scattered through the land to tell us where the plague exists, and how to control and remove it.

And I appeal to all who have the prosperity of the country at heart, and realize the full value of that great industry with which our animals are connected, to provide for the increase of colleges of this description, and to the liberal endowment and encouragement of this institution whose commencement exercises we have been called here to witness.

Every humane master, who regards his horse and his dog with a kindly eye; every wise and affectionate father, who knows the value of healthful food to his family; every lover of a wide-spread and generous prosperity for man and beast, cannot set too high value upon this modern endeavor to establish and develop and apply the best theory and practice of veterinary science, and to furnish the healing art in all its best forms to the dumb animals upon whom we so constantly depend.

